# Recent Advances in Two-Dimensional Metallic MXenes as High-Performance Saturable Absorbers

**DOI:** 10.3390/nano16120733

**Published:** 2026-06-12

**Authors:** Xin Xiong, Jiancheng Zheng, Jiahao Huang, Yuxian Yang, Xiyan Huang, Chibiao Liu

**Affiliations:** Fujian Key Lab of Agriculture IOT Application, School of Information Engineering, Sanming University, Sanming 365004, China; 20240865149@fjsmu.edu.cn (X.X.); 20240865218@fjsmu.edu.cn (J.H.); yangyx@fjsmu.edu.cn (Y.Y.); xyhuang@fjsmu.edu.cn (X.H.)

**Keywords:** MXene, saturable absorber, passive mode-locking, ultrashort pulses, nonlinear optics, two-dimensional materials, high-performance mode-locking materials

## Abstract

Passively mode-locked lasers, as essential tools for generating ultrashort pulses, have found widespread applications in industrial manufacturing, optical communications, biomedical imaging, and fundamental scientific research. Saturable absorbers serve as the key components governing the performance of such laser systems. Conventional saturable absorber materials, including semiconductor saturable absorber mirrors, carbon nanotubes, and graphene, however, suffer from inherent limitations in operational wavelength range, damage threshold, and environmental stability. In recent years, two-dimensional transition metal carbides and nitrides, known as MXenes, have emerged as a promising class of materials to address these challenges. Their unique metallic conductivity, broadband saturable absorption, ultrafast carrier dynamics, excellent thermal management capability, and versatile chemical tunability offer unprecedented opportunities for advanced saturable absorber applications. This review systematically summarizes the recent progress of MXene-based saturable absorbers, with an emphasis on their distinctive advantages in extending the mode-locked wavelength range, enhancing output pulse stability, and increasing the optical damage threshold. Furthermore, strategies for performance optimization through surface terminal group engineering, defect modulation, and heterostructure design are discussed in depth. Finally, the future prospects and key challenges toward industrial implementation of MXenes in ultrafast photonics are outlined, aiming to stimulate further advancements in high-performance ultrafast laser technology.

## 1. Introduction

Passively mode-locked lasers are core devices for ultrashort pulse generation and have played vital roles in micro/nano manufacturing, ultrahigh-speed optical communications, biomedical imaging, and precision spectroscopy [1]. The saturable absorber (SA) is a key nonlinear component that directly governs the pulse duration, energy, and long-term stability of the output [2]. After decades of development, conventional SAs—including semiconductor SA mirrors (SESAMs), carbon nanotubes, and graphene—have been widely adopted, yet their inherent limitations have become bottlenecks for further progress. Specifically, SESAMs have narrow spectral response, require complex epitaxial fabrication, and are costly [3,4]; the nonlinear response of carbon nanotubes is strongly chirality-dependent, and aggregation during large-scale fabrication often degrades performance [5,6]; graphene, despite its ultrabroad spectral response, has an intrinsically low modulation depth (typically <2%) and is prone to thermal damage under high-power pumping [7,8]. Thus, the search for novel SA materials offering broadband response, high modulation depth, ultrafast recovery, high damage threshold, and environmental stability remains a persistent goal.

Since the first synthesis of MXenes by Gogotsi et al. in 2012 [9,10,11], these layered materials have attracted broad interest in energy, catalysis, and optoelectronics [12]. Their metallic conductivity (10^3^–10^4^ S/cm), broad spectral response (UV to mid-infrared), sub-picosecond carrier relaxation, and high nonlinear absorption coefficients make them well-suited for high-performance SAs [13,14,15,16,17,18]. In 2017, Jhon et al. demonstrated a femtosecond mode-locked fiber laser using metallic Ti_3_CN MXene, pioneering the field [19,20]. Subsequently, Jiang et al. characterized the broadband nonlinear optical response of Ti_3_C_2_T_x_ (800–1800 nm) and achieved 159 fs mode-locked pulses, laying a solid foundation [17,21].

Research on MXene-based SAs has grown explosively. The material family has expanded from Ti_3_C_2_T_x_ and Ti_3_CN to many other compositions, including V_2_CT_x_, Nb_2_CT_x_, Ti_2_CT_x_, Mo_2_Ti_2_C_3_T_x_, Ta_4_C_3_T_x_, V_4_C_3_, and Nb_4_C_3_, and the operating wavelength range now covers 1 to 3 µm [22,23,24,25,26,27]. Establishing quantitative correlations between MXene structure and nonlinear optical performance has become a key issue [22,28], which helps to understand trade-offs and guide device optimization [29,30,31].

This review differs from previous summaries by focusing on three aspects. First, we emphasize cross-comparative analysis of quantitative structure–property relationships. Second, beyond static optimization strategies, we discuss the emerging concept of dynamic regulation, whereby external stimuli could tune the saturable absorption during device operation. Third, we address industrial challenges—oxidation, non-saturable loss, and mid-infrared mode-locking—and outline pathways including machine learning and scalable fiber integration. The review is organized as follows: Section 2 covers material fundamentals; Section 3, synthesis and integration; Section 4, performance cross-comparison; Section 5, optimization strategies; Section 6, challenges and future outlook; Section 7, conclusions. Using ultrashort pulse generation, high-energy output, and novel pulse states as guiding threads, we cross-compare results and analyze enhancement mechanisms, aiming to provide a reference for the further development of high-performance MXene-based ultrafast lasers.

## 2. Material Fundamentals and Nonlinear Optical Properties of Metallic MXenes

### 2.1. Crystal Structure and Typical Material Systems

MXenes are two-dimensional materials obtained by selectively etching the “A” layer (typically Al, Si, etc.) from their layered ternary ceramic precursors, the MAX phases [32], with a general formula of M_n+1_X_n_T_x_, where M represents an early transition metal (e.g., Ti, V, Nb, Mo), X is carbon and/or nitrogen, and T_x_ denotes surface terminations (–O,–OH, –F). This unique etching process endows MXenes with an accordion-like layered structure and rich surface chemistry. The weak van der Waals forces between layers facilitate their exfoliation into few-layer or monolayer nanosheets, providing a structural basis for optical fiber integration.

**Figure 1:**
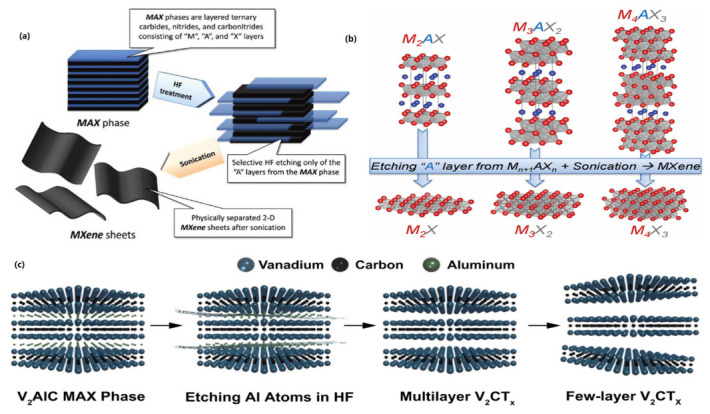
Schematic illustration of the conversion from MAX phases to MXenes. (**a**) Selective etching of “A” layers by HF followed by ultrasonic exfoliation (Reprinted from Ref. [9] with permission. © 2012 ACS). (**b**) Layered structures of M_2_AX, M_3_AX_2_, and M_4_AX_3_ MAX phases (Reprinted from Ref. [35] with permission. © 2014 Wiley). (**c**) Atomic-scale etching and exfoliation process of V_2_AlC to few-layer V_2_CT_x_ nanosheets (Reprinted from Ref. [36] under CC BY 4.0). In subfigure (**b**), arrows indicate the direction of the process; different colored circles represent M, A, and X atoms, respectively.

Members of the MXene family with metallic conduction have attracted considerable attention for nonlinear optics. First-principles calculations reveal that the metallic nature of MXenes such as Ti_3_CN—as opposed to the semi-metallic nature of graphene—underlies their broadband saturable absorption [33,34]. Figure 1a illustrates the complete process: MAX phases are selectively etched with HF to remove the “A” layers, followed by ultrasonic exfoliation to yield accordion-like multilayer MXenes and single-/few-layer nanosheets [9]. Figure 1b shows the layered stacking of three typical MAX phases—M_2_AX, M_3_AX_2_, and M_4_AX_3_—reflecting that the thickness of the M_n+1_X_n_ layer increases with n [35]. Figure 1c uses V_2_AlC as an example to show, at the atomic scale, the transformation of Al atoms etched by HF into multilayer V_2_CT_x_ and further exfoliated into few-layer V_2_CT_x_ nanosheets [36]. Together, these panels summarize the synthesis route, structural evolution, and material examples, providing an intuitive basis for understanding MXenes’ layered characteristics and their potential for optical fiber integration [37].

Table 1 summarizes the fundamental physical properties and nonlinear optical performance parameters of several representative metallic MXenes that have been widely investigated or have recently emerged in the field of ultrafast photonics. As can be seen from the table, the electrical conductivities of these MXene systems generally lie in the range of 10^2^–10^4^ S/cm [16,38], and their work functions fall within the interval of 4.3–5.5 eV [24,39], demonstrating favorable metallic conduction characteristics that provide the necessary electronic structure basis for broadband saturable absorption. With regard to surface terminations, most systems feature –O and –OH as the dominant terminating groups, and their relative proportions significantly influence the density of states near the Fermi level and the carrier relaxation behavior. In terms of modulation depth, different MXene systems exhibit strong composition dependence and wavelength selectivity—for example, Ti_3_C_2_T_x_ has established a well-defined performance benchmark in the 1–2 μm region [39,40,41], while MXenes show excellent modulation depth potential in the mid-infrared region (e.g., Ti_3_C_2_T_x_ at 2.95 μm) [42]. Furthermore, each system displays distinct advantages: the ultrahigh damage threshold of V_2_CT_x_ makes it suitable for high-power lasers [43,44], the ultrafast relaxation time (on the picosecond scale) of Nb_2_CT_x_ provides the kinetic basis for GHz high-repetition-rate applications [16,45], and ordered bimetallic systems such as Ti_4_C_3_T_x_ MXene demonstrate potential for dual-wavelength mode-locking [41,46]. This rich diversity of material choices and their complementary performance characteristics lay the foundation for the application-oriented optimization discussed in subsequent chapters.

Beyond the Ti-based MXenes that dominate the current literature, other compositions such as Sc-, Ta-, and Mo-based MXenes, as well as high-entropy MXenes, have recently emerged as potential candidates for nonlinear optical applications. Theoretical studies suggest that Sc_2_C-based MXenes exhibit semiconductor-like band structures that could be tuned for specific wavelength ranges, while Mo_2_Ti_2_C_3_T_x_ already demonstrates exceptional modulation depth in the mid-infrared (Table 1). High-entropy MXenes, which incorporate multiple transition metals into the M layers, offer an additional degree of freedom for tailoring electronic and optical properties, although their synthesis remains challenging and their nonlinear optical performance has not yet been systematically evaluated. Nemani et al. first reported the synthesis of high-entropy MXenes TiVNbMoC_3_T_x_ and TiVCrMoC_3_T_x_, demonstrating that the high-entropy strategy can significantly expand the compositional variety of the MXene family to further tune their electronic, electrochemical, and thermal properties [57]. A comprehensive comparison of the structure–property relationships across these less-studied MXene families is still lacking, but they hold promise for overcoming some limitations of Ti-based systems, particularly in terms of oxidation resistance and bandgap engineering.

Table 1 compiles key properties of representative MXene SAs, but several issues warrant critical reflection. The wide tunability of composition and termination offers clear advantages [13,16,24], yet the large spread in reported conductivities for the same material—Ti_3_C_2_T_x_ ranges from 1500 to >24,000 S/cm—reveals a lack of standardized processing [13,58]. Similar inconsistencies exist for modulation depth; values for V_2_CT_x_ at 1.55 µm vary from 1.6% to ~50% depending on measurement configuration [26,43,47,59]. Such variability undermines direct cross-study comparison and calls for community-agreed characterization protocols [28,40,58]. Another subtle point is the inherent trade-off behind individual metrics. Ta_4_C_3_T_x_ has high third-order polarizability but unquantified modulation depth [54]; V_2_CT_x_ offers a high damage threshold (~70 mJ/cm^2^) but may suffer from slower relaxation [43,60]; Nb_2_CT_x_ delivers ultrafast recovery (~37 fs) at the cost of modest modulation depth (~13%) [45,50]. No single composition is universally optimal; material selection should be guided by specific laser requirements. Future work must prioritize reproducible synthesis and systematic structure-property mapping under controlled conditions [58,61].

### 2.2. Intrinsic Advantages as SAs

In metallic MXenes such as Ti_2_CT_x_, the band structure exhibits a high density of states near the Fermi level and no intrinsic bandgap [17,62]. This metallic nature allows free-carrier intraband transitions to be excited regardless of incident photon energy, giving rise to absorption from the visible to the mid-infrared. Experimentally, Ti_2_CT_x_ shows pronounced saturable absorption from 800 nm to 2.8 μm [32,63,64], and related MXenes such as Ti_3_C_2_T_x_ also exhibit broadband saturable absorption from 800 nm to 1800 nm and even 2000 nm [17,62]. These findings provide a theoretical basis for “single-material compatibility with multiple wavelength bands”, fundamentally overcoming the bandgap limitations of semiconductor SAs.

The high density of electronic states at the Fermi level (e.g., up to 1.2 × 10^21^ states/(eV·cm^3^) for Ti_3_C_2_T_x_) ensures that a large number of carriers can be rapidly excited to the conduction band under intense photoexcitation, thereby producing a pronounced Pauli blocking effect. This directly translates into large modulation depth and high third-order nonlinear absorption coefficients. For instance, Jiang et al. measured the effective nonlinear absorption coefficient of Ti_3_C_2_T_x_ to be on the order of ~10^−21^ m^2^/V^2^ [65,66], while the ordered bimetallic Mo_2_Ti_2_C_3_T_x_ exhibits a modulation depth as high as 40% in the mid-infrared region [17,53].

**Figure 2:**
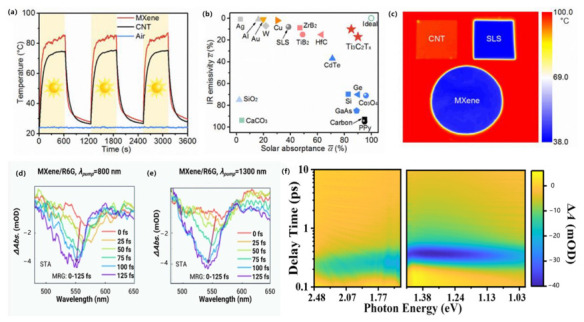
Photothermal conversion performance and ultrafast carrier dynamics of metallic MXenes. (**a**–**c**) Photothermal heating capability and spectral absorption comparison (Reprinted from Ref. [67] with permission. © 2021 Wiley). (**d**,**e**) Transient absorption spectra of Ti_3_C_2_ at 800 nm and 1300 nm excitation (Reprinted from Ref. [68] under CC BY 4.0). (**f**) Ultrafast relaxation map across visible to near-infrared (Reprinted from Ref. [33] with permission. © 2022 ACS).

The above three advantages—broadband response, high modulation depth, and ultrafast relaxation—are all rooted in the metallic electronic structure of MXenes. Figure 2 multi-dimensionally demonstrates the intrinsic advantages of MXenes as saturable absorbers, spanning from photothermal conversion and spectral absorption to ultrafast carrier dynamics: Figure 2a–c respectively present the temperature variation curve under standard solar irradiation, a comparison of the absorption and emission rates of various materials, and infrared thermal images, confirming the excellent photothermal conversion performance and broadband absorption characteristics of MXenes [67]; Figure 2d,e show the transient absorption spectra of Ti_3_C_2_ under 800 nm and 1300 nm excitation, where a pronounced negative differential absorption signal is observed over a broad spectral range, and the response region shifts toward longer wavelengths as the excitation wavelength is redshifted, reflecting the universality and tunability of non-resonant excitation [68]; the transient absorption map in Figure 2f further reveals that Ti_3_C_2_ exhibits a uniform and ultrafast relaxation signal (~tens of femtoseconds) across the visible to near-infrared region, whereas conventional materials display wavelength-dependent slow relaxation characteristics [33]. The above results experimentally corroborate the unique advantage of metallic MXenes—“single-material compatibility with multiple wavelength bands”—fundamentally overcoming the bandgap limitations of conventional semiconductor SAs.

The strong M–X bonds and metallic conductivity give MXenes excellent thermal stability and heat dissipation [39,69]. MXenes have ultrahigh damage thresholds (e.g., V_2_CT_x_ ~70 mJ/cm^2^ [43,70]), nearly an order of magnitude higher than graphene [26]. Because of their high thermal conductivity, V_2_CT_x_ rapidly dissipates heat from hot spots across the film via 2D diffusion channels, suppressing thermal damage and ensuring stable high-power operation [39,43,71].

### 2.3. Comparison with Conventional Saturable Absorbers

To contextualize the advantages of MXenes among the rapidly expanding family of two-dimensional materials, it is instructive to directly compare their key performance metrics with those of conventional saturable absorbers. Table 2 provides a side-by-side assessment of MXene (taking Ti_3_C_2_T_x_ as a representative) against semiconductor saturable absorber mirrors (SESAMs), graphene, carbon nanotubes (CNTs), black phosphorus (BP), and transition metal dichalcogenides (TMDs). The comparison covers spectral range, modulation depth, recovery time, damage threshold, environmental stability, fabrication complexity, cost, and integration flexibility. As summarized in the table, MXenes offer an attractive combination of broadband operation (visible to mid-IR), moderate to high modulation depth (up to 88% in composites), ultrafast recovery (sub-picosecond to few picoseconds), and high damage threshold (e.g., ~70 mJ/cm^2^ for V_2_CT_x_) [17,47], while their main drawback—ambient oxidation—can be mitigated by encapsulation. This cross-material benchmark highlights where MXenes excel and where further improvements are still needed.

In summary, MXenes offer a combination of broadband spectral response, high modulation depth, ultrafast recovery, and high damage threshold that collectively outperform or rival conventional SAs. Compared with SESAMs, MXenes are more cost-effective and spectrally flexible; versus graphene, they provide an order-of-magnitude higher modulation depth; and relative to CNTs, they avoid chirality-dependent performance variations. Black phosphorus shares MXenes‘ broadband mid-IR response but suffers from rapid ambient degradation, whereas TMDs cover a narrower wavelength range and generally exhibit lower modulation depths. The main remaining challenge for MXenes—ambient oxidation—can be mitigated by encapsulation and composite strategies, as discussed in Section 5.4 and Section 6.1.

Beyond the metrics summarized in Table 2, practical considerations such as long-term reliability, fabrication yield, device-to-device reproducibility, and environmental robustness are equally critical for real-world applications. SESAMs benefit from mature semiconductor processing, offering high reproducibility and lifetimes of several years, but at a high cost and with limited spectral flexibility [3,4]. Graphene and carbon nanotubes suffer from batch-to-batch variations; for nanotubes, chirality control remains a persistent challenge [5,6]. MXenes, while promising, still face difficulties in achieving consistent surface termination and layer number across large areas, which directly affects the reproducibility of modulation depth and saturation intensity. Their environmental stability, although improved by encapsulation, remains inferior to that of SESAMs and graphene [7,8]. Concerning the related question of whether graphite or exfoliated turbostratic graphite could be used for similar optical applications: graphite is a semimetal with a finite density of states at the Fermi level, and thick graphite films exhibit broadband absorption but lack the strong evanescent-field interaction that makes atomically thin MXene nanosheets attractive for fiber-integrated SAs. Moreover, the difficulty of producing uniform, few-layer turbostratic graphite films and the absence of tunable surface chemistry make graphite less versatile than MXenes for tailoring nonlinear optical responses. Comprehensive comparisons of conventional and 2D material SAs can be found in recent reviews, which further highlight the trade-offs among modulation depth, bandwidth, stability, and cost [2,22,74]. Therefore, although graphite may function as a saturable absorber in certain configurations, it does not offer the same combination of solution processability, surface functionalization, and broadband modulation depth that MXenes provide. With the material foundations established, the next section discusses synthesis and fiber integration.

## 3. Preparation and Integration Strategies for MXene-Based SAs

### 3.1. Scalable Synthesis Methods

Efficient synthesis methods are a prerequisite for practical MXene applications. The choice of method influences layer number, lateral size, termination, and defect density, which govern the nonlinear optical performance.

HF etching, the classical approach, yields high-quality MXenes but its high toxicity and abundant –F terminations limit green industrialization [35,75,76]. Even at low HF concentrations, the product forms –OH-rich surfaces, which are thermodynamically stable but cause uncertainties in electrochemical performance [77].

To overcome the safety hazards and limited termination tunability of concentrated HF, milder etching systems have been actively developed. One widely adopted approach uses a mixture of LiF and HCl, which generates HF in situ and simultaneously intercalates Li^+^ ions, facilitating delamination and yielding high-quality Ti_3_C_2_T_x_ with fewer defects and a higher proportion of –O/–OH terminations [35,58]. Similar fluoride-salt/HCl systems (e.g., NaF, KF) and ammonium bifluoride (NH_4_HF_2_) have also been successfully used to etch MAX phases, offering safer handling and enabling the introduction of –Cl, –Br, or –I terminations depending on the acid chemistry [78]. More recently, a solvent-free gas-phase etching method has been demonstrated, where volatile fluorine-containing species are used to selectively remove the A layer without any liquid by-products, representing a promising route for green and scalable MXene production [79]. These alternative etching routes not only improve process safety but also provide finer control over the surface chemistry and defect density, both of which critically influence the nonlinear optical performance of MXene-based SAs.

Electrochemical exfoliation, a fluoride-free route, uses an electric field to drive ion intercalation, yielding products with high –OH/–O termination ratios and good conductivity, and enabling continuous large-area film preparation for industrialization [80,81,82]. The MILD strategy, which replaces ultrasonication with manual shaking, produces large-area, low-defect MXene nanosheets without introducing additional defects and has become the mainstream method for high-quality films [75,82].

Liquid-phase exfoliation is simple and low-cost. With appropriate solvents and sonication, high-yield MXene nanosheets can be prepared. The method is compatible with solution-based processing (spin-coating, spray-coating, photodeposition) and is the mainstream for laboratory-scale fabrication. For instance, Wu et al. used photodeposition to deposit Ti_3_C_2_T_x_ onto a microfiber, achieving a high-performance SA [28,83,84]. Mixed solvent systems (e.g., water-ethanol or water-DMSO) reduce surface tension, improve exfoliation efficiency, and avoid toxicity and high boiling points of conventional organic solvents [84,85].

Molten salt-assisted synthesis has also been explored. Ahmad et al. prepared Ti_2_C MXene using a molten fluoride salt and applied it for mode-locking at 2 µm [86]. This method opens a new route to high-purity MXene compositions. Eutectic salt systems (e.g., LiCl-KCl) have also been investigated; their low melting points and strong Lewis acidity enable efficient exfoliation of MAX phases at relatively low temperatures, reducing energy consumption and pollution [87,88,89].

### 3.2. Surface Termination Engineering

Surface terminations serve as a bridge linking the atomic structure of MXenes to their macroscopic properties. Generally, –F terminations are considered detrimental to performance, as they introduce strongly electronegative defects on the Ti layers, leading to electron localization and thus significantly reducing the electrical conductivity [90,91]. In contrast, –OH and –O terminations, owing to their intrinsic polar nature, can effectively enhance the density of electronic states near the Fermi level and facilitate the formation of electron transport channels, thereby strengthening the nonlinear response and metallic properties of the materials [92,93].

The distinct influences of –O, –OH, and –F terminations originate from their different electronic and chemical characteristics. Density functional theory calculations have shown that –O terminations maintain a high density of states at the Fermi level, preserving the metallic band structure that underlies broadband saturable absorption [61,94]. In contrast, –F terminations, owing to their strong electronegativity, tend to localize electrons and reduce the density of states near the Fermi level, effectively widening the bandgap and thereby weakening the nonlinear optical response. –OH terminations exhibit an intermediate behavior, retaining a moderate density of states while introducing polaronic states that facilitate carrier transport [61,94]. These electronic structure differences directly translate into distinct carrier dynamics. The ultrafast relaxation times observed in –OH/–O terminated MXenes arise from efficient electron–phonon coupling enabled by the polar nature of these surface groups, which is essential for high-repetition-rate mode-locking [24,60]. By contrast, –F terminations can act as scattering centers, potentially prolonging the relaxation time and compromising the ultrafast response required for femtosecond pulse generation. In terms of chemical stability, –O terminations form stronger bonds with the underlying metal atoms, providing better passivation against oxidative degradation, whereas –F terminations are more readily desorbed under humid conditions, accelerating material deterioration [78,95]. Consequently, the modulation depth, recovery time, and long-term stability of MXene-based SAs can be effectively tailored by controlling the relative proportions of –O, –OH, and –F terminations through post-annealing or plasma treatment [61,96].

To achieve precise control over such chemical states, researchers have developed various post-processing techniques. For instance, post-annealing (thermal treatment under an inert atmosphere such as Ar or N_2_) can drive the desorption or rearrangement of unstable –F groups on the surface, converting them into more stable and more conductive –OH or –O groups, thereby significantly enhancing electrical conductivity and chemical stability [78,95]. In addition, plasma treatment offers a more selective approach: by tuning the plasma power and gas atmosphere composition (e.g., NH_3_/Ar mixture), –OH or –O terminations can be selectively introduced or enriched without disrupting the layered structure of MXenes, enabling fine regulation of surface polarity and hydrophilicity [95,97].

Such precise tailoring of surface chemical states allows not only modulation of the Fermi level position in MXenes, but also effective control over key parameters such as carrier relaxation time, photoluminescence intensity, and dielectric constant, providing a strong physical foundation for high-performance flexible sensors, optoelectronic devices, and high-frequency communication materials. Relevant details will be elaborated in Section 5 in conjunction with experimental data.

### 3.3. Fiber Integration Structures

#### 3.3.1. Tapered/Micro-Nano Fiber

**Figure 3:**
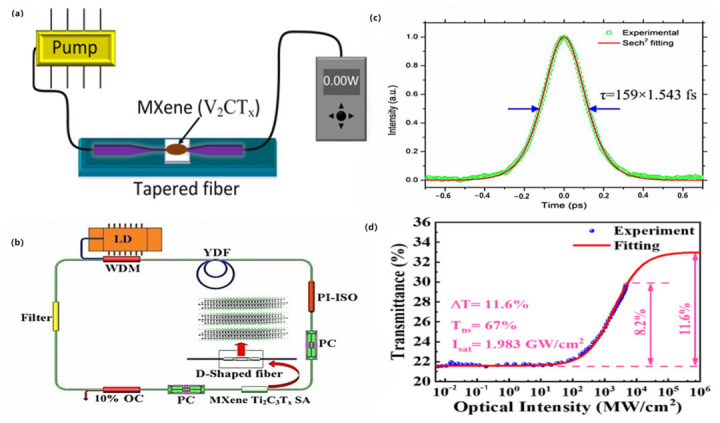
Tapered fiber integration and nonlinear optical performance of Ti_3_C_2_T_x_ MXene. (**a**) Evanescent wave coupling scheme (Reprinted from Ref. [43] under CC BY 4.0). (**b**) Ring cavity mode-locked laser setup (Arrows indicate the direction of laser propagation in the cavity; Reprinted from Ref. [17] with permission. © 2018 Wiley-VCH). (**c**) Autocorrelation trace fitted with sech^2^ function (Reprinted from Ref. [17] with permission. © 2018 Wiley-VCH). (**d**) Nonlinear transmittance fitting curve (Reprinted from Ref. [40] under CC BY 4.0).

The tapered fiber represents the current “gold standard” for achieving high modulation efficiency with MXenes in research settings. By finely stretching the optical fiber, the core diameter is gradually reduced to the micrometer or even sub-micrometer scale, thereby generating a strong evanescent field at the core surface. After MXene nanosheets are deposited on the tapered region, the interaction length between light and matter is significantly extended, resulting in a greatly enhanced modulation depth; moreover, because the light–matter interaction occurs outside the original core, the insertion loss is extremely low. This structure has been widely employed in femtosecond pulse lasers. For example, harmonic mode-locking up to the 36th order (218.4 MHz repetition rate) has been realized using Ti_3_C_2_T_x_ MXene, with a modulation depth as high as 30% and a pulse duration down to 850 fs [83,98]. This combination of long interaction length and high modulation efficiency makes it a preferred approach for generating high-power femtosecond pulses in the laboratory. Figure 3 comprehensively illustrates, using Ti_3_C_2_T_x_ as an example, the working principle, device implementation, and nonlinear optical performance characterization of the tapered fiber integration structure. Figure 3a shows a schematic of the evanescent wave coupling between the tapered fiber and MXene: pump light is injected into the tapered fiber region, and the MXene coated on the taper waist (V_2_CT_x_ is taken as an example in the figure) couples with the propagating optical field through the evanescent field, enabling nonlinear modulation of the light intensity [43]; Figure 3b presents the architecture of an MXene saturable absorber-based fiber ring mode-locked laser, which comprises a laser diode (LD), a wavelength-division multiplexer (WDM), a ytterbium-doped fiber (YDF), a polarization-independent isolator (PI-ISO), a polarization controller (PC), and an MXene saturable absorber (Ti_2_CT_x_ is taken as an example in the figure), forming a complete ring cavity for generating ultrashort pulses [17]; Figure 3c displays the autocorrelation trace fitted by a hyperbolic secant squared (sech^2^) function [17]; and Figure 3d provides the nonlinear transmittance fitting curve measured by the Z-scan or dual-arm balanced detection method [40].

#### 3.3.2. D-Shaped Fiber

D-shaped fibers are fabricated by precision polishing to remove a portion of the cladding, forming a planar region that allows the evanescent field of the fiber to directly interact with MXene nanosheets deposited on the flat surface. Owing to the planar geometry, MXene films can be deposited with high uniformity via spin-coating or drop-casting techniques, and the heat dissipation efficiency at the metal/glass interface is enhanced, together with excellent mechanical stability. Research by Ahmad et al. demonstrated that depositing a Ti_3_C_2_-PVA film (thickness ~ 63.6 μm) onto a D-shaped fiber enabled the generation of soliton pulses as short as 2.21 ps at a center wavelength of 1557.63 nm, and exhibited improved stability compared with conventional point-contact methods [99,100]. This structure is particularly suitable for high-power laser systems requiring long-term operation, as its planar design effectively suppresses thermal accumulation within the fiber and mitigates environmental perturbations.

#### 3.3.3. Hollow-Core Anti-Resonant Fiber

In contrast to conventional evanescent-field interaction, hollow-core anti-resonant fibers (HC-ARFs) achieve low-loss transmission through specific structural designs such as thin-walled tube arrays. Recent studies have demonstrated that filling MXene Ti_3_C_2_T_x_ into the outer cladding tubes of an HC-ARF enables the high-intensity optical field distribution within the hollow-core structure to interact nonlinearly with the MXene, thereby achieving a modulation depth as high as 30% and an insertion loss as low as 3.5 dB [101]. This structure opens a new avenue for enhancing light–matter interaction efficiency, exhibiting outstanding optical performance particularly in the mid-infrared (2 μm) region.

#### 3.3.4. Etched Fiber and Fiber End-Face Film

Etched fibers are fabricated by chemically transforming the fiber profile into a parabolic-like shape (e.g., via HF etching), thereby creating a broader evanescent field region. This well-established and easily standardized process is suitable for mass production. For instance, by immersing an etched fiber into a liquid MXene dispersion, ultrafast mode-locking with a pulse width of ~800 fs has been achieved [102].

**Figure 4:**
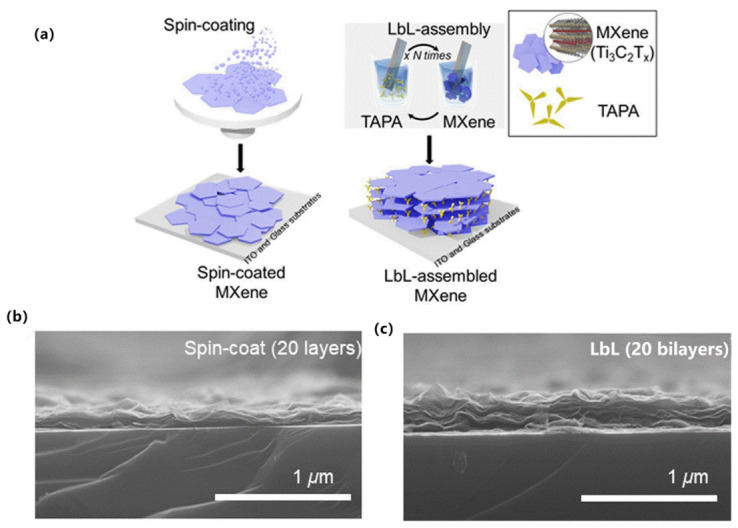
Preparation methods and end-face integration of MXene films. (**a**) Spin-coating vs. layer-by-layer assembly processes. (**b**) Cross-sectional SEM images showing layered structures. (**c**) Fiber end-face before and after MXene deposition (Reprinted from Ref. [104] under CC BY-NC 4.0).

Fiber end-face films, on the other hand, are realized by directly attaching an MXene thin film (such as a Ti_3_C_2_T_x_–PVA composite film) onto the fiber facet. The structure is extremely simple, facilitating convenient “plug-and-play” operation. This configuration is frequently employed for rapid prototype validation and industrial applications, as it circumvents the need for complex fiber drawing or polishing processes [103]. In the preparation of fiber end-face films, the choice of film deposition technique significantly influences the device performance. As shown in Figure 4a, spin-coating and layer-by-layer (LbL) assembly represent two typical methods. The cross-sectional SEM images in Figure 4b compare the resulting film morphologies: the LbL method (20 bilayers) produces a more regular and ordered layered structure than spin-coating (20 layers), indicating better thickness uniformity and reduced scattering loss. Figure 4c shows an optical micrograph of a fiber patch cord end-face before and after MXene deposition, where the uniformly coated film enables a “plug-and-play” SA integration structure [104].

Table 3 compares five MXene fiber integration structures across interaction strength, insertion loss, mechanical stability, thermal management, and industrialization potential. Distinct trade-offs exist among the different schemes. Tapered and hollow-core fibers offer high interaction strength and modulation depth, making them preferred platforms for laboratory ultrashort pulse generation [28,31,102,105]. Tapered fibers increase interaction area by ~300× via evanescent field coupling, with insertion loss as low as 0.87 dB, modulation depth of 6.4%, and damage threshold up to 19.34 GW/cm^2^ [44,100]. Hollow-core fibers achieve 30% modulation depth and 3.5 dB insertion loss at 2 µm by filling MXene into cladding tubes [106,107]. D-shaped fibers feature excellent mechanical and thermal stability: their planar geometry facilitates heat conduction, maintaining mode-locking at 1500 mW pump power with non-saturable loss as low as 3%, making them suitable for high-power industrial lasers [44,99,108,109]. Etched fibers offer a mature, standardized process with low insertion loss; immersion in liquid MXene enables ~800 fs pulses, giving them high industrialization potential for mass production [39,41,110]. Fiber end-face films are the most compact, with typical insertion loss ~3 dB and modulation depth tunable from 1.8% to 19.1%; their “plug-and-play” convenience is ideal for rapid prototyping and education [7,111,112,113]. MXenes’ own high thermal conductivity further enhances device stability [20,40,106,109]. Based on these preparation and integration techniques, the following section evaluates device performance.

## 4. Performance Characteristics and Cross-Comparative Analysis of Core Mechanisms

Building on the materials and devices discussed in the preceding sections, this chapter provides a cross-comparative integration and analysis of the core performance of MXene SAs in mode-locked and Q-switched lasers. Rather than organizing the discussion by operating wavelength bands, we focus on three core performance dimensions in the field of ultrafast lasers, namely, ultrashort pulse generation, high-energy/high-power output, and novel pulse states.

### 4.1. The Core of Ultrashort Pulse Generation

Transient absorption spectroscopy studies show that carrier relaxation in MXenes is dominated by electron–phonon coupling, with a fast process (τ_1_) on the order of tens to hundreds of femtoseconds. Notably, the τ_1_ of Nb_2_CT_x_ has been reported to be as low as ~37 fs (i.e., approximately 37 fs), a value significantly faster than those of graphene and most TMDs, providing the key kinetic basis for achieving high repetition rates and narrow pulse widths [17,60]. This sub-picosecond fast process is primarily attributed to the strong electron–phonon interaction induced by the surface terminations of MXenes, such as –OH groups [24,60].

**Figure 5:**
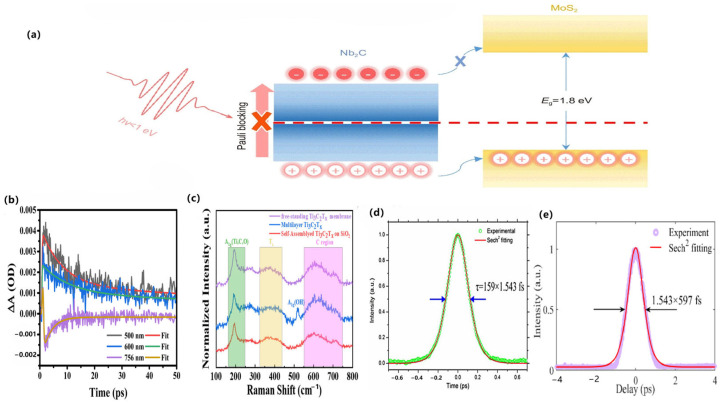
Correlation between ultrafast carrier dynamics and ultrashort pulse generation in MXenes. (**a**) Charge transfer in Nb_2_C/MoS_2_ heterostructure (Reprinted from Ref. [24] under CC BY 4.0). (**b**) Transient absorption kinetics (Reprinted from Ref. [16] under CC BY-NC 4.0). (**c**) Raman spectrum of Ti_3_C_2_T_x_ (Reprinted from Ref. [40] under CC BY 4.0). (**d**) 159 fs pulse autocorrelation (Reprinted from Ref. [17] with permission. © 2018 Wiley-VCH). (**e**) 316 fs pulse autocorrelation (Reprinted from Ref. [47] with permission. © 2019 Optica Publishing Group).

Further compressing the pulse duration through materials engineering is a current research focus. Extensive experimental evidence has demonstrated that Ti_3_C_2_T_x_ with a high proportion of –OH terminations can significantly enhance nonlinear absorption [24]. Jiang et al. first achieved a pulse output of 159 fs in the 1.55 μm band [17], and subsequently Wu et al. further compressed the pulse duration to 104 fs by employing micro-nano fiber integration [47]. In the 1 μm band, Sun et al. obtained 316 fs pulses in an all-solid-state laser. The realization of the above ultrashort pulses is rooted in the ultrafast carrier relaxation dynamics and tunable electronic structure of MXenes. Figure 5 systematically illustrates this correlation from the two dimensions of microscopic mechanism and macroscopic performance: The enhanced nonlinear absorption mechanism in the Nb_2_C/MoS_2_ heterostructure is schematically shown in Figure 5a, where photogenerated electrons transfer from MoS_2_ to Nb_2_C, suppressing recombination and enhancing Pauli blocking [24]. Figure 5b shows the transient absorption kinetics at probe wavelengths of 500 nm, 600 nm, and 756 nm, all exhibiting sub-picosecond relaxation times [16]. Figure 5c presents the Raman spectrum of Ti_3_C_2_T_x_ in the range of 100–800 cm^−1^, where the A_1_g peak at approximately 197 cm^−1^ reflects structural ordering and the influence of surface terminations [40]. Representative mode-locked pulse autocorrelation traces are displayed in Figure 5d,e: Jiang et al. achieved a pulse duration of 159 fs (after sech^2^ fitting with a factor of 1.543) at 1.55 µm [17], while Sun et al. obtained 316 fs pulses (Gaussian fitting) at 1.05 µm with a spectral width of 4.26 nm [47].

Reducing MXene dimensions to quantum dots or a few layers is a common strategy to exploit quantum confinement and shorten pulses [126]. However, Jhon et al. found that even highly stacked Ti_3_C_2_T_x_ (hundreds of layers) can generate 897 fs pulses at 1.9 µm [16]. First-principles calculations attribute this to a unique interlayer coupling effect mediated by surface functional groups, which allows electronic states in thick films to still participate efficiently in photoexcitation and relaxation [16,28]. This finding challenges the “fewer-layer-better” paradigm and reveals a distinct property of MXenes: unlike traditional two-dimensional materials, stacked MXene structures can still achieve efficient nonlinear modulation. This opens the possibility of simplifying fabrication without stringent layer-number control.

Furthermore, the slow carrier relaxation time in some MXenes is relatively long (>100 ps). Although this is beneficial for Q-switched pulse generation, it may limit their application in mode-locking for generating even shorter femtosecond pulses [60,127]. How to tailor the carrier relaxation pathways through termination and defect engineering is key to achieving superior pulse quality in the future.

Table 4 summarizes the sub-picosecond/femtosecond mode-locked pulse performance achieved with SAs based on different MXene systems. As can be clearly seen from the table, MXene-based SAs have realized ultrashort pulse outputs ranging from sub-picosecond to hundreds of femtoseconds in multiple wavelength bands, and the further compression of pulse duration is closely correlated with the structural characteristics of the materials and the integration strategies employed. In the 1.55 μm telecommunication band, Ti_3_C_2_T_x_, benefiting from a high proportion of –OH terminations and the evanescent field enhancement effect of micro-nano fibers, has successively achieved record short pulse durations of 159 fs [39] and 104 fs [47], with the latter currently representing the optimal level for MXene-based SAs in this band. In the 1 μm band, Ti_3_C_2_T_x_ has delivered 316 fs pulses on an all-solid-state laser platform [128], expanding the application scenarios of MXene SAs. Notably, a highly stacked Ti_3_C_2_T_x_ structure with hundreds of layers still achieved mode-locked output of 897 fs in the 1.91 μm band [129], thereby revising the conventional empirical belief that “fewer layers always yield better performance.” Meanwhile, Nb_4_C_3_, owing to its ultrabroadband response, realized dual-wavelength picosecond mode-locking at 1.56/1.93 μm in a single device [130]. In addition, Ti_3_CNT_x_, as the pioneering validation material, produced short pulses of 660 fs [131], and V_2_C demonstrated mode-locking with a high signal-to-noise ratio of 92 dB [132] as well as an ultrashort pulse output of 72 fs [26] through surface plasmon resonance enhancement effects. These results, achieved via different technical routes, collectively verify the broad spectral applicability and diversified optimization space of the MXene family in the field of ultrashort pulse generation.

The pulse performance in Table 4 highlights both the capabilities and the unresolved challenges of MXene SAs. The shortest pulse (104 fs) is achieved not by the highest modulation depth but by optimized microfiber integration, showing that device engineering can outweigh intrinsic material properties [17,47,83]. Highly stacked Ti_3_C_2_T_x_ yields 897 fs pulses, contradicting the “fewer-layer-better” assumption. Interlayer coupling preserves nonlinear response even in thick films, a behavior distinct from graphene or TMDs and recently confirmed in layer-dependent studies [16,20,129,133]. This offers a practical advantage—simplified fabrication—but its generality across other MXenes remains unknown. A more pressing issue is the large variability: the same Ti_3_C_2_T_x_ gives pulse durations from 104 fs to 897 fs depending on thickness and cavity design, making fair comparison difficult [17,20,47,129]. Moreover, while sub-200 fs pulses are routine at 1.55 µm, the 3 µm mid-IR region remains limited to Q-switching, with no femtosecond mode-locking reported [42,53,134]. This gap points to a need for faster recovery or better damage resistance in the mid-IR. The observed layer-number-dependent transition from reverse saturable to saturable absorption offers a potential tuning route [133]. Overall, systematic studies separating intrinsic performance from measurement artifacts are urgently needed, along with a concerted push toward mid-IR femtosecond lasers [40,58].

### 4.2. Thermal Management and Damage Threshold Analysis for High-Performance Laser Output

MXenes’ high electrical conductivity allows rapid transport of Joule heat across the film, and their high thermal conductivity ensures efficient heat dissipation. Together, these attributes endow MXenes with ultrahigh damage thresholds: V_2_CT_x_ ~70 mJ/cm^2^ and Ti_3_CNT_x_ >50 mJ/cm^2^, nearly an order of magnitude higher than graphene [25,43].

In the realm of high-power solid-state lasers, Sun et al. employed few-layer Ti_3_C_2_T_x_ as an SA in a 1 µm Yb:KYW all-solid-state laser and successfully achieved stable mode-locked output with an average power of 0.77 W and a pulse duration of 316 fs, fully demonstrating the high-power application potential of MXenes on solid-state laser platforms [128]. In terms of high-energy pulse generation, MXenes have likewise exhibited notable advantages: Zhang et al. obtained Q-switched pulses with a single-pulse energy as high as 20.8 µJ using a Ti_3_C_2_T_x_ SA in a 2 µm Ho:YLF laser [135]; Wei et al., in a 2.8 µm mid-infrared Er:ZBLAN fiber laser, realized Q-switched operation with an average power of 1.09 W and a single-pulse energy of 13.93 µJ by integrating Ti_3_C_2_T_x_ onto the fiber end-face, achieving a modulation depth as high as 33.2% [136]. The realization of the aforementioned high-energy Q-switched results benefits from the high optical damage threshold conferred by the excellent metallic electrical conductivity and efficient thermal management capability of MXenes. Furthermore, progress has also been made in further enhancing mode-locked pulse energy through composite structure design—for instance, compositing Ti_3_C_2_T_x_ with a polymer matrix or constructing heterostructures has enabled mode-locked pulse energy output on the order of tens of nanojoules in the 1 µm and 1.55 µm bands [137], offering a viable optimization pathway for the development of high-energy femtosecond sources. At present, in the mid-infrared region, especially around 3 µm, MXene SAs have mainly achieved high-energy Q-switched operation, whereas a clear breakthrough in mode-locked femtosecond pulses has yet to be realized. This may be related to the low photon energy in this band, the requirement for higher nonlinear response, and the greater complexity of intracavity dispersion management [25]. In the future, exploring novel MXenes with higher modulation depths, such as Mo_2_Ti_2_C_3_T_x_, together with optimized cavity design, will be key to achieving mid-infrared femtosecond mode-locking.

### 4.3. Broadband Tunability and Mid-Infrared Band Expansion Outperform the Spectral Limitations of Conventional Materials

The metallic gapless band structure of MXenes endows them with the unique potential for ultrabroadband response spanning from the visible to the mid-infrared, which has been verified in multiple wavelength bands.

Ahmad et al. constructed a broadly tunable Q-switched laser operating in the 2 μm region by coating a microfiber with Ti_3_C_2_T_x_. In a thulium-doped fiber laser (TDFL), they achieved continuously tunable output over 155 nm (1895–2050 nm), fully demonstrating the broadband modulation capability of MXenes in the “eye-safe” wavelength band [138].

**Figure 6:**
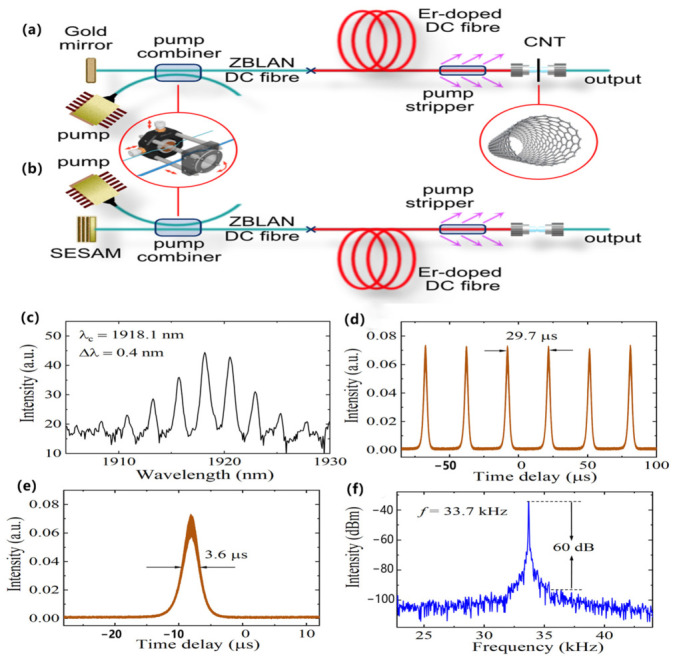
Schematic of fiber laser configurations and Q-switched output performance using different saturable absorbers. (**a**,**b**) CNT-based and SESAM-based Er:ZBLAN fiber lasers (Arrows indicate the direction of laser propagation; Reprinted from Ref. [145], licensed under CC BY 4.0.). (**c**–**f**) Output spectrum, pulse train, single pulse profile, and RF spectrum of a Ag/Ti_3_C_2_-based Q-switched laser at 2 µm (Reprinted from Ref. [146] under CC BY 4.0).

MXenes have demonstrated stable Q-switched operation in the mid-infrared (2.7–3 µm) in both solid-state and fiber lasers. In solid-state lasers, Fan et al. first achieved Q-switching at 2.95 µm using an MXene SA [42]; Hao et al. later extended the wavelength to 2.73 µm [139], and Wang et al. reported Q-switching at 3 µm in a Ho:ZBLAN fiber laser [140]. In fiber lasers, Guo et al. employed an ordered double-transition-metal Mo_2_Ti_2_C_3_T_x_ SA in a 2.8 µm Er:ZBLAN laser, achieving a modulation depth of 40%—far exceeding that of conventional 2D materials such as MoS_2_ (~5%)—and validating the potential of MXenes for efficient mid-IR Q-switching [134,141,142]. Jung et al. used a V_4_C_3_ MXene SA in a 2.7 µm Er:ZBLAN laser, delivering high-energy pulses (1.59 µJ) with tunable width and stable repetition rate [53,143]. Ti_3_C_2_T_x_ has also been successfully applied in watt-level mid-IR fiber lasers [144]. Compared with SESAMs, which require complex fabrication and are costly [53], MXenes offer simple processing, tunable properties, and high modulation depth, making them attractive for mid-infrared fiber lasers. Figure 6a,b show schematic diagrams of Er:ZBLAN lasers using CNT- and SESAM-based SAs, respectively, for comparison. Figure 6c–f present the output spectrum, pulse train, single-pulse profile, and RF spectrum of a Ag/Ti_3_C_2_-based Q-switched laser at 2 µm, confirming stable operation with narrow linewidth, uniform train, and high signal-to-noise ratio [145,146].

### 4.4. Nonlinear Dynamics of Novel Pulse States Enter a New Stage

In the area of high-repetition-rate harmonic mode-locking, Feng et al. successfully achieved harmonic mode-locking up to the 36th order using Ti_3_C_2_T_x_, elevating the pulse repetition rate to 218.4 MHz. This is attributed to the strong support of the ultrafast carrier relaxation characteristics of MXenes for the stable operation of high-repetition-rate pulse trains, demonstrating the application potential of MXenes in high-repetition-rate light sources [83,147]. Regarding dissipative soliton resonance (DSR), the high modulation depth and low non-saturable loss of MXenes enable precise balancing of nonlinearity and loss in dispersion-managed cavities, thereby achieving DSR pulse output with tunable pulse width and extremely high energy, opening up application prospects in fields such as photonic radar that require flexible pulse shapes [1]. Furthermore, the polarization-independent property of MXenes (with extremely low polarization-dependent loss) makes them a preferred material for generating stable vector solitons [17]; by finely adjusting the pump power and intracavity dispersion, MXene-based lasers can further generate complex pulse states such as soliton molecules, providing a convenient experimental platform for investigating soliton interactions and nonlinear dynamics [148]. To further improve the performance metrics discussed above, Section 5 reviews various materials engineering strategies.

## 5. Performance Optimization Strategies

To address the challenges of MXenes in terms of wavelength-specific response, non-saturable loss, and stability, researchers have developed a series of materials engineering strategies to achieve targeted optimization of their overall performance.

### 5.1. Electronic Structure Modulation Strategies

Electronic structure modulation, including termination engineering and elemental doping, optimizes MXene performance by modifying the density of states near the Fermi level.

**Figure 7:**
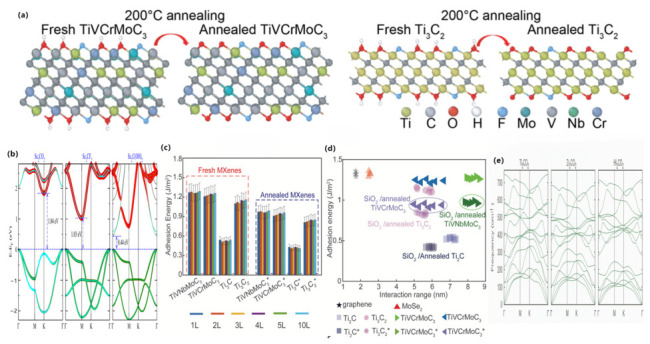
Optimization effects of electronic structure modulation strategies on MXenes. (**a**) Annealing treatment principle (Reprinted from Ref. [150] under CC BY-NC-ND 4.0). (**b**) Band structures of Sc_2_CT_x_ with different terminations (Reprinted from Ref. [94] with permission. © 2016 APS). (**c**,**d**) Adhesion energy comparison (Reprinted from Ref. [150] under CC BY-NC-ND 4.0). (**e**) Band structures and density of states of M_2_CO_2_ (M = Ti, Zr, Hf) (Reprinted from Ref. [152] with permission. © 2013 Wiley).

As discussed in Section 3.2, tuning surface terminations via annealing or plasma treatment enhances MXene conductivity and nonlinear absorption [61,96,149]. Beyond termination engineering, elemental doping (N, S, Cl) is another strategy to modulate the electronic structure. Figure 7 illustrates these optimization effects. Figure 7a shows that annealing at 200 °C removes –F and replaces them with –OH/–O, improving conductivity and nonlinear absorption [150]. Figure 7b compares band structures of Sc_2_CT_x_ with different terminations: –O maintains metallicity and high density of states, while –F and –OH widen the bandgap [94]. Figure 7c,d compare adhesion energies of pristine and annealed MXenes with other 2D materials; annealed MXenes show higher adhesion energies, indicating stronger interfacial binding [150]. Finally, Figure 7e presents band structures and density of states of M_2_CO_2_ (M = Ti, Zr, Hf). The choice of M element significantly affects the metallic characteristics; Ti_2_CO_2_ exhibits the highest density of states at the Fermi level [151,152]. 

Introducing heteroatoms (such as N, S, Cl) into the MXene lattice is another efficient strategy for modulating its electronic structure, namely elemental doping. For example, nitrogen doping (N-doping) can not only provide additional electrons or holes but also introduce new active sites or modify the work function, thereby enhancing electrical conductivity and thermal stability [153,154,155]. The doping process can occur through in-lattice substitution (e.g., C replaced by N), surface adsorption, or functional group replacement, with these distinct doping mechanisms corresponding to different modifications of the physicochemical properties [153,156].

### 5.2. Morphology and Dimensionality Control

This approach primarily encompasses two directions: MXene quantum dots and few-layer/stacked structures. When the size falls below the exciton Bohr radius, the quantum confinement effect leads to discrete energy levels and a significant opening of the bandgap, thereby markedly enhancing the nonlinear absorption coefficient (β) [89,157]. Moreover, the presence of surface terminations (–F, –OH, =O, –Cl) on quantum dots further modulates the carrier dynamics, with experimental results demonstrating that the carrier rise time can reach the sub-picosecond scale [16]; this is the core advantage of quantum dot modulation. On the other hand, conventional few-layer structures, owing to their lower surface roughness, effectively reduce scattering loss and exhibit excellent nonlinear optical response [22]. Notably, even highly stacked MXenes can maintain efficient nonlinear modulation under certain conditions due to interlayer coupling effects, as discussed in detail in Section 4.1 [15,129].

### 5.3. Interfacial Synergistic Enhancement

Compositing MXenes with other materials boosts performance through interfacial effects. MXene/2D heterojunctions expand the spectral response range and enhance nonlinear optical performance. For example, combining MXenes with graphene exploits the zero-bandgap structure of graphene and the high carrier density of MXenes to achieve broadband optical absorption modulation from the visible to the mid-infrared [17]. Meanwhile, van der Waals heterostructures formed by MXenes and transition metal dichalcogenides (TMDs, such as MoS_2_) can effectively suppress carrier recombination through interfacial charge transfer and band alignment, significantly enhancing nonlinear optical processes such as second-harmonic generation or third-harmonic generation, thereby exhibiting a “1 + 1 > 2” synergistic effect [24,51].

**Figure 8:**
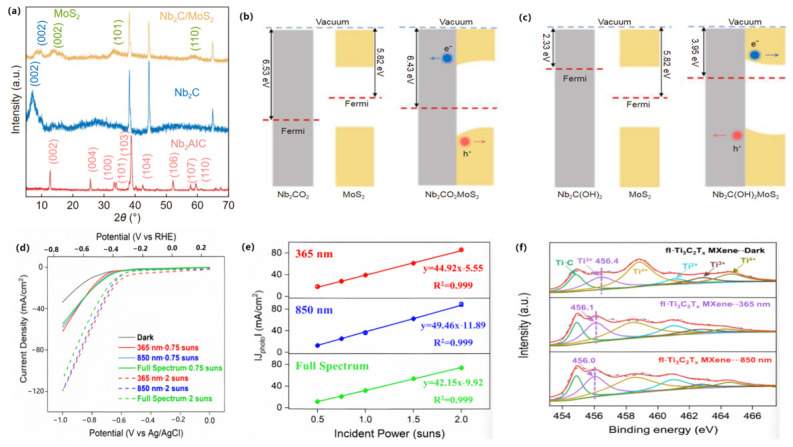
Interfacial characterization and photoelectrochemical performance of MXene-based heterostructures. (**a**) XRD patterns of Nb_2_C/MoS_2_ heterostructure (Reprinted from Ref. [24] under CC BY-NC 4.0). (**b**,**c**) Band alignment before and after contact (Reprinted from Ref. [24] under CC BY-NC 4.0). (**d**–**f**) LSV curve, photocurrent vs. intensity, and in situ XPS of few-layer Ti_3_C_2_T_x_ (Reprinted from Ref. [160] with permission. © 2025 ACS).

MXene/metal nanocomposites represent a key strategy. Yuan et al. prepared Ti_3_C_2_T_x_/Au nanocomposites via an ultrasound-assisted method. The LSPR effect of Au nanoparticles couples with intrinsic MXene plasmons, forming a strongly coupled plasmon mode that enhances the local optical field, increases nonlinear modulation depth, reduces saturation intensity, and achieves dual-wavelength mode-locked output at 1030.6 nm and 1551.4 nm [158,159]. Wang et al. further exploited the intrinsic plasmon resonance of V_2_C MXene for high-performance mode-locking or Q-switching, demonstrating the broad potential of MXene plasmon-coupled modulation [132]. Figure 8 provides experimental and theoretical support for interfacial synergistic enhancement using the Nb_2_C/MoS_2_ van der Waals heterostructure. Figure 8a shows XRD patterns of Nb_2_C, Nb_2_AlC, MoS_2_, and the heterostructure. Figure 8b,c show the band alignment of the Nb_2_CO_2_/MoS_2_ heterointerface before and after contact [24]. Figure 8d presents the linear sweep voltammetry curve of few-layer Ti_3_C_2_T_x_ in 0.05 mol/L H_2_SO_4_, evaluating its electrochemical catalytic activity. Figure 8e shows the photocurrent density versus light intensity, reflecting the carrier separation and transport capability. Figure 8f compares in situ XPS spectra of Ti 2p orbitals in the dark and under 365 nm and 850 nm illumination [159]. These characterizations reveal the interfacial synergistic enhancement mechanisms from electrochemistry, photocatalysis, and surface chemistry perspectives.

### 5.4. Macroscopic Engineering Adaptation

To address the issues of easy agglomeration and poor film-forming ability of MXene nanosheets, compositing them with optically transparent polymer matrices (such as PVA, PMMA, and PI) is a commonly adopted strategy [161,162,163].

Polyvinyl alcohol (PVA), owing to its excellent optical transmittance and hydrophilicity, can form a dense network structure between MXene flakes, effectively suppressing layer restacking and reducing scattering loss α_ns_ [161,164,165]. Moreover, the PVA matrix imparts high flexibility and environmental tolerance to the composite film, enabling it to maintain stable optoelectronic properties during bending or stretching processes [64,162,163].

Polymethyl methacrylate (PMMA), as an inorganic/organic composite matrix, possesses an outstanding optical transparency window and chemical inertness, providing mechanical support while maintaining low optical absorption. When doped with MXenes, it can be used for interfacial passivation in high-efficiency perovskite solar cells, significantly improving device stability [166].

Polyimide (PI), by virtue of its exceptional high-temperature resistance and mechanical strength, is suitable for applications under high-temperature or harsh environmental conditions. MXene/PI composite films not only retain flexibility but also exhibit excellent electrothermal conversion efficiency and long-term cycling stability [167].

In summary, the selection of the matrix should comprehensively consider the optical transmission window, mechanical strength, and thermal stability to meet the requirements of specific application scenarios such as flexible wearable devices or high-temperature environments [162,163].

Beyond improving processability, polymer matrices also provide a degree of protection against ambient oxidation, though their effectiveness varies with the polymer type, film thickness, and environmental conditions. For example, PVA forms a dense hydrogen-bonded network that can slow the diffusion of oxygen and water vapor, but its hydrophilic nature may actually promote moisture absorption under high humidity, partially offsetting the barrier effect [165]. PMMA, being hydrophobic and chemically inert, offers better moisture resistance, but its relatively low glass transition temperature (≈105 °C) limits its use in high-power laser systems where local heating is significant. PI, with its exceptional thermal stability (up to ≈400 °C) and low gas permeability, provides the most robust protection among the three, yet its processing requires organic solvents and higher curing temperatures, which may not be compatible with all MXene formulations [162,163]. A key point is that the primary function of these matrices in most reported works remains the improvement of film uniformity, flexibility, and adhesion, rather than the deliberate optimization of gas-barrier properties [164]. Consequently, the protection afforded by a pristine polymer coating is often insufficient for long-term device operation under ambient conditions, especially against photo-induced degradation. This is why dedicated encapsulation strategies—such as multilayer barriers, inert atmosphere storage, or atomic layer deposition of Al_2_O_3_—are discussed separately in Section 6.1 as more effective means to ensure environmental stability [168]. Nonetheless, the combination of a polymer matrix with a subsequent encapsulation layer can synergistically enhance both the mechanical integrity and the oxidative resistance of MXene SAs, a strategy that merits further systematic investigation.

It is noteworthy that the above strategies all belong to “static” optimization prior to device fabrication. However, the unique surface chemical activity and layered structure of MXenes also offer the possibility of “dynamic” regulation during device operation.

Table 5 summarizes the four main performance optimization strategies for MXene SAs, including their regulatory targets, implementation pathways, performance gains, and current limitations. Termination engineering directly modulates the density of electronic states and conductivity. Removing –F and enriching –OH via annealing or plasma treatment improves modulation depth and nonlinear coefficients [47,83,149]. For example, –OH-terminated MXene shows stronger reverse saturable absorption at 800 nm (βeff = 113 ± 3.2 cm GW^−1^), and vacuum annealing increases conductivity from 85 S·m^−1^ to 241 kS·m^−1^ [169,170]. Dimensionality control reduces MXenes to 0D quantum dots, exploiting quantum confinement to enhance nonlinear absorption and accelerate relaxation. 0D MXene quantum dots exhibit broadband response (540–1550 nm), a nonlinear absorption coefficient β ≈ −(11.24 ± 0.14) × 10^−2^ cm/GW, a relaxation time of ≈1.28 ps, and have enabled 170 fs pulses in Er/Yb co-doped fiber lasers [17,40,88,157]; however, scalable synthesis and stacking mechanisms remain challenging. Heterostructure/composite construction uses interfacial charge transfer and field enhancement to broaden the response range and synergistically boost nonlinearity. MXene/Au nanocomposites achieve enhanced modulation depth at 1.03/1.55 μm (13.6% for AuNP-PVA films), and Mo_2_Ti_2_C_3_T_x_ reaches 40% at 2.8 μm [41,53,171,172,173]; weak interfacial bonding and complex fabrication are key bottlenecks. The composite matrix strategy improves film-forming ability and mechanical stability. MXene/PVA nanofibers show n_2_ = −8.08 × 10^−6^ cm^2^/W and β = −0.014 cm/W at 532 nm, and MXene/CNC composites enhance dispersion stability [110,174,175,176,177]; however, low polymer thermal conductivity and poor dispersibility at high concentrations limit performance. These strategies are not mutually exclusive; multi-dimensional synergistic regulation is often required, leading to the dynamic regulation concept discussed in Section 5.5 [28,41,178].

### 5.5. Exploration of the Potential for Dynamic Regulation of Smart Responsive MXenes

The aforementioned optimization strategies all focus on the static tailoring of the structure and properties of MXenes—that is, predefining their nonlinear optical response through materials engineering before device fabrication. However, the unique two-dimensional layered structure and excellent surface chemical activity of MXenes endow them with tunable electronic properties that can respond reversibly to external stimuli (such as electric fields, optical fields, gas molecules, and ion intercalation) [41,183]. The metallic MXenes discussed throughout this review have no bandgap at the Fermi level, but their electronic structure—most notably the density of states near the Fermi level—remains highly sensitive to surface terminations, interlayer spacing, and external perturbations. For semiconducting MXene variants, a composition-dependent bandgap provides an additional dimension for dynamic control. Thus, the concept of “tunable bandgap” is more relevant to semiconducting MXenes, while for metallic MXenes the focus is on modulating the Fermi-level density of states and carrier dynamics. Theoretical predictions and experimental studies have shown that the saturable absorption performance of MXenes is highly sensitive to the incident light intensity, surface functional groups, and interlayer spacing [184]. This property opens up new possibilities for developing “smart SAs”—SA devices capable of dynamically regulating their nonlinear response during operation.

It should be emphasized, however, that most of the dynamic regulation concepts discussed in this section remain at the proof-of-concept or theoretical stage. To date, only a few experimental demonstrations have been reported in the context of MXene SAs. For example, Cu ion intercalation has been shown to reversibly expand the interlayer spacing of Ti_3_C_2_T_x_, modulating its electronic structure and, consequently, its optical properties [148]. The photothermal effect in Ti_3_C_2_ nanosheets has also been characterized, demonstrating stable cycling and tunable temperature response, which could potentially be exploited for photo-induced reversible modulation [185]. Even in these cases, however, the effects have been characterized in isolation rather than integrated into a functioning, reconfigurable ultrafast laser cavity. Concepts such as gate-voltage tuning of saturable absorption, gas-induced reversible modulation, and self-optimization via photothermal structural evolution have not yet been experimentally realized in working SA devices. Thus, the following discussion is intended primarily to stimulate future research and to highlight promising directions, rather than to summarize mature technologies.

The excellent electronic conductivity (up to ~10^4^ S/cm) and ion intercalation capability of MXenes make them an ideal platform for electrochemical modulation [24]. Gate voltage or electrochemical ion intercalation (Li^+^, Na^+^, K^+^, Cu^2+^) can reversibly tune the Fermi level and interlayer spacing [95]. Such regulation is predicted to induce orders-of-magnitude changes in carrier concentration, thereby significantly altering the saturable absorption threshold and modulation depth [95,161]. Experimentally, CTAB-based cationic intercalation expands the interlayer spacing from 12.6 Å to 21.4 Å, facilitating rapid ion diffusion and active site exposure [74]; Cu ion intercalation exhibits a unique “guest/host redox” coupling mechanism, modulating the oxidation states of both Cu and Ti_3_C_2_T_x_ during charging [183]. Surface termination regulation can directly “switch on/off” the nonlinear optical absorption of MXenes [40,186]. For example, varying the –O/–F/–OH ratio creates higher occupancy states near the Fermi level, enhancing conductivity and nonlinear optical efficiency [95,187]. Although theoretical models based on two-level atomic systems have explained the optical nonlinearity of MXenes in the vis–NIR region [28], this concept remains largely at the exploratory stage. Its potential application value should not be overlooked: it may enable “on-demand switching” within a single laser cavity—dynamically selecting between femtosecond mode-locking and high-energy Q-switching, or adaptively adjusting the SA response to input intensity [110,184]. Several works have confirmed the stability of MXene SAs in fiber lasers; for instance, a Ti_3_C_2_T_x_-based mode-locked ytterbium-doped fiber laser achieved 100 fs pulses, laying a foundation for dynamically regulated devices [39,110].

The surface terminations of MXenes (especially –OH and –O) are highly sensitive to gas molecules such as NH_3_, NO_2_, and H_2_O. Upon adsorption, charge transfer and surface dipole changes alter the conductivity and work function. DFT calculations indicate that –OH-terminated MXenes strongly adsorb acidic molecules, whereas –O terminations affect electron scattering and carrier mobility [188]. This property enables nonlinear regulation coupled with gas and humidity sensing. Gas exposure triggers complex nonlinear charge transport modulation in Ti_3_C_2_T_x_, and intercalation of gas molecules expands the interlayer spacing, disrupting vertical charge transport and reducing conductivity [189]. These sensing characteristics have been validated: Ti_3_C_2_T_x_ gas sensors detect VOCs at 50–100 ppb with a signal-to-noise ratio two orders of magnitude higher than other 2D materials [190]; alkalization (Na^+^ intercalation, increased O/F ratio) gives a ~60-fold response change over 11–95% RH and 28.87% response to 100 ppm NH_3_ [191]. MXene resistance increases linearly with humidity from 15% to 80% RH (up to 26-fold change), with response time superior to commercial sensors [192]. MXenes also exhibit saturable and reverse saturable absorption, applicable to passive optical switches, ultrafast lasers, and photonic diodes [193]. Their nonlinear absorption coefficients can reach 10^−13^ esu, enabling Q-switched and mode-locked pulse lasers [194]. Because of their broadband response, high nonlinearity, and ease of integration, MXenes have become primary SA materials in ultrafast photonics [110]. Integrating an MXene SA into a gas environment could realize gas/humidity-gated nonlinear optical modulation, potentially leading to pulsed laser sensors whose output parameters (repetition rate, pulse width) vary with gas concentration, enabling optical-readout gas sensing [171].

MXene materials themselves possess strong optical absorption and highly efficient photothermal conversion capabilities, a feature that holds promise for achieving photo-induced reversible modulation. According to reported studies, MXenes exhibit strong optical absorption in the NIR region, with an internal photothermal conversion efficiency close to 100% [195]. Under high-power laser irradiation, the localized photothermal effect can induce subtle changes in the interlayer spacing or partial desorption/rearrangement of surface terminations. The photothermal conversion mechanism of MXenes is attributed to the LSPR effect—when the frequency of photons absorbed by MXene matches the intrinsic frequency of electrons on the surface of the metallic particles, resonance occurs at the metal–dielectric interface, and the induced coherent charge oscillations dissipate into the surrounding medium through lattice scattering vibrations, thereby raising the temperature [195]. Although this effect has traditionally been regarded as a precursor to photo-induced damage, if it can be precisely controlled within the sub-damage threshold range, such photo-induced structural evolution could potentially be exploited as a “self-calibration” or “self-optimization” mechanism, enabling the SA response characteristics to adapt automatically to the pump power. Studies have found that the photothermal effect of Ti_3_C_2_ nanosheets can be fine-tuned by adjusting the nanosheet concentration or laser intensity, and that Ti_3_C_2_ nanocomposites exhibit no significant changes in temperature–time curves or peak shape after four heating–cooling cycles, demonstrating excellent photothermal stability [196]. This outstanding photothermal conversion performance also renders them promising for applications such as photo-driven actuators [195].

The dynamic regulation strategies proposed in this subsection extend the study of MXene SAs from the static optimization of “materials engineering” to a new paradigm of dynamic intelligent regulation at the “device physics” level. Although the relevant research is still in its infancy, the organic integration of these strategies with the aforementioned static approaches, such as termination engineering and dimensionality control, is expected to give rise to a new generation of multifunctional, reconfigurable ultrafast photonic devices [171,193]. With the continuous development of low-dimensional optical materials, research on the application of MXenes in photonics is steadily deepening. Further exploration of their nonlinear optical response and dynamic regulation mechanisms will provide new technological pathways for the development of integrated devices combining ultrafast lasers and gas sensing [171,194]. Despite these optimization strategies, several critical challenges remain, as outlined in Section 6.

## 6. Challenges and Future Perspectives

Although MXene-based SAs have made considerable progress, several core challenges remain on the path toward industrial application.

### 6.1. Current Core Challenges

**Figure 9:**
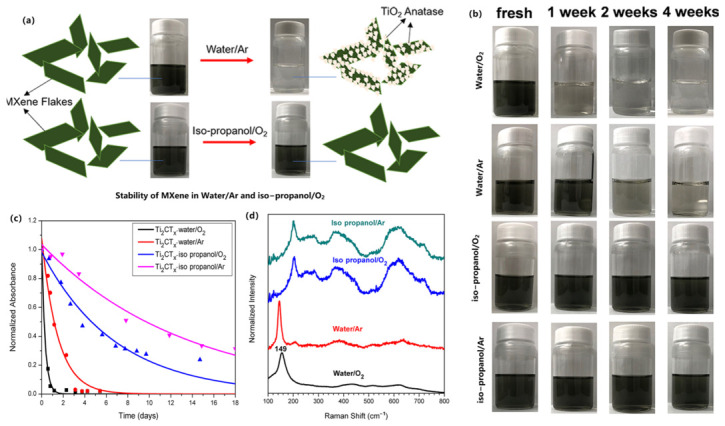
Oxidation stability and degradation mechanisms of Ti_3_C_2_T_x_ MXene under different environmental conditions. (**a**) Normalized absorbance decay over time in water/isopropanol under O_2_ or Ar. (**b**) Raman spectra evolution showing anatase TiO_2_ formation. (**c**) Normalized absorbance of Ti_3_C_2_T_x_ dispersed in water and isopropanol under O_2_ or Ar atmospheres as a function of time. (**d**) Raman spectra of Ti_3_C_2_T_x_ after different storage periods under water/O_2_, water/Ar, isopropanol/O_2_, and isopropanol/Ar conditions (Reprinted from Ref. [199] with permission. © 2019 ACS).

First, MXene material systems, particularly Ti_3_C_2_T_x_, are susceptible to oxidative degradation in air and water, which constitutes a prominent issue concerning their long-term stability [197,198,199]. Although surface terminations can provide a certain degree of passivation, the specific oxidation pathways and failure mechanisms under high-power, high-humidity, and high-temperature operating conditions still require in-depth in situ investigation [168,200,201]. Developing effective encapsulation techniques or intrinsically more stable MXene compositions (such as Ta_4_C_3_T_x_) represents a potential solution. As shown in Figure 9a, the normalized absorbance of Ti_3_C_2_T_x_ MXene decreases most rapidly when dispersed in water under an O_2_ atmosphere, while Ar protection or dispersion in isopropanol significantly retards the degradation [199]. The Raman spectra in Figure 9b reveal that under water/O_2_ conditions, the characteristic peaks of MXene (region i) gradually weaken with time, while peaks assigned to anatase TiO_2_ (region ii) emerge and grow, directly revealing the oxidation pathway from Ti atoms to TiO_2_ [199], providing evidence for understanding the failure mechanisms and evaluating the effectiveness of encapsulation strategies [199].

Under continuous laser pumping, local heating and high peak intensities accelerate chemical changes even in encapsulated films—a phenomenon known as photo-induced degradation. Several interdependent degradation pathways directly affect key SA parameters. One prominent pathway is the conversion of surface terminations: –OH and –O groups gradually transform into less conductive –F species under sustained irradiation, reducing the density of states near the Fermi level and thus diminishing the nonlinear absorption coefficient. This effect, combined with the general oxidation kinetics of MXenes in aqueous environments, is highly sensitive to pH and temperature, with alkaline conditions and elevated temperatures accelerating degradation [201]. Simultaneously, the oxidation of Ti atoms leads to anatase TiO_2_ nanocrystals, which consume the active MXene phase and introduce scattering centers that increase non-saturable loss. The role of surface chemistry in this process has been systematically investigated; McIntosh et al., using in situ Raman spectroscopy and microwave conductivity measurements, showed that the oxidation process is heavily influenced by the synthesis route and initial surface chemistry of Ti_3_C_2_T_x_, with fluoride and oxyfluoride groups playing a pivotal role in stabilizing the anatase phase [202]. Complementary studies on laser-induced damage have identified distinct Raman spectral markers for the transformation from the original MXene lattice to TiO_2_ nanoparticles, providing a spectroscopic basis for real-time monitoring of photothermal degradation [203]. Restacking of delaminated flakes, accelerated by local photothermal effects, further increases insertion loss and degrades modulation depth. A progressive decrease in electrical conductivity weakens thermal dissipation, creating a positive feedback loop where higher local temperatures accelerate further degradation. In this context, intrinsically more stable MXene compositions, such as Ta_4_C_3_T_x_ and Nb_4_C_3_T_x_, exhibit significantly higher oxidation resistance due to their stronger M–X bonds, offering a route toward more robust SA devices [64,168]. Collectively, these processes result in a gradual reduction in modulation depth, an increase in saturation intensity, and ultimately mode-locking failure. A systematic understanding of how these degradation mechanisms depend on pump wavelength, repetition rate, pulse energy, and ambient conditions is still lacking. Accelerated aging tests under controlled environments are urgently needed to establish reliable lifetime predictions.

To address this oxidation challenge, various encapsulation and stabilization strategies have been developed. As described in Section 5.4, embedding MXenes in polymer matrices (e.g., PVA, PMMA, PI) provides an effective physical barrier against oxygen and moisture [162,163,164,165]. Another approach involves atmosphere and solvent control: storing MXene dispersions or fabricated SAs in inert atmospheres (Ar or N_2_, <1 ppm O_2_) or using deoxygenated anhydrous solvents (e.g., ethanol or isopropanol) instead of water significantly retards oxidation [197,199]. More advanced techniques include surface chemical passivation, such as coating a thin Al_2_O_3_ layer via atomic layer deposition (ALD) or grafting antioxidant molecules onto the MXene surface [168]. Additionally, intrinsically more stable MXene compositions, such as Ta_4_C_3_T_x_ and Nb_4_C_3_T_x_, exhibit higher oxidation resistance due to stronger M–X bonds, albeit sometimes with compromised nonlinear optical performance [54,158,168]. Despite these promising strategies, standardized accelerated aging tests and long-term reliability data for MXene SAs under real laser operating conditions are still lacking, and establishing such protocols is a critical step toward industrial translation.

Second, significant batch-to-batch and inter-laboratory variations in the lateral size, number of layers, and termination concentration of MXenes result in substantial fluctuations in device performance [32,58]; methods such as HF etching face inherent difficulties in precisely controlling the layer number and surface functional groups. Establishing unified standards for material preparation and characterization is therefore an urgent priority [25,146].

A closely related but often overlooked challenge is the lack of standardization in the measurement and reporting of nonlinear optical parameters. The modulation depth, saturation intensity, and even the sign of nonlinear absorption extracted from Z-scan or balanced detection experiments depend heavily on a range of experimental conditions: film thickness (which can vary from sub-10 nm to several micrometers), substrate effects, laser pulse width (femtosecond versus picosecond), wavelength, beam profile, and the fitting model employed (e.g., the two-level saturable absorption model versus a three-level model that includes excited-state absorption). For example, the same Ti_3_C_2_T_x_ film can yield a modulation depth ranging from 11% to over 50% solely because of variations in thickness and measurement configuration [40,47]. Such variability makes it nearly impossible to reliably compare the intrinsic nonlinearity of different MXene compositions or to judge the true performance advantage of a new material based on literature data.

To mitigate this problem, the community would benefit from adopting common reference materials (e.g., a standard graphene film or a commercial SESAM) measured under identical conditions, and from openly reporting all relevant experimental parameters, including film thickness, laser pulse width, peak intensity range, beam spot size, and fitting details. Moreover, the development of standardized test protocols—possibly through interlaboratory round-robin tests—is urgently needed to enable fair benchmarking and to accelerate the transition of MXene SAs from laboratory research to practical industrial applications [40,58].

Moreover, non-saturable loss (α_ns_) represents a key factor limiting laser efficiency [25,39], and its origins are complex, involving lattice defects, surface terminations, interface states, and agglomeration. Currently, effective means for the atomic-level tracing and quantitative control of α_ns_ remain lacking [21,146]. In addition, the current understanding of the mechanisms underlying mid-infrared mode-locking is still insufficient. Despite the notable achievements in mid-infrared Q-switching, femtosecond mode-locking in the 3 μm band has yet to be realized, which may be attributed to the low photon energy in this region, the relatively weak nonlinear response of the materials, and the complexity of intracavity dispersion management [42,204,205]. How to translate the advantage of high modulation depth into mode-locked femtosecond pulses constitutes the central scientific question for the next stage. Finally, some MXenes exhibit relatively long slow relaxation times (>100 ps), which, although favorable for Q-switching, may hinder the generation of even shorter femtosecond pulses [16,206]. Precisely tailoring the “fast” and “slow” carrier relaxation pathways through termination, defect, and dimensionality engineering to achieve optimal kinetic response under specific application scenarios remains in need of more in-depth investigation [207,208].

### 6.2. Future Directions

**Figure 10:**
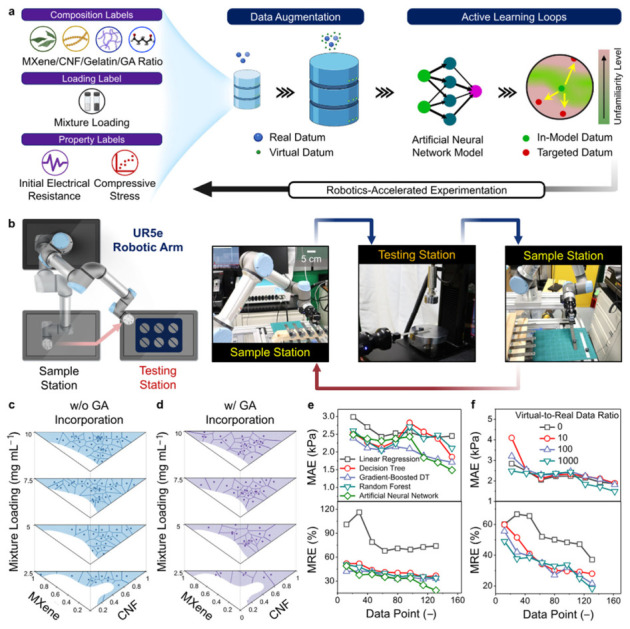
Automated experimentation and machine-learning-assisted design framework for MXene composites. (**a**) Material composition labels. (**b**) UR5e robotic arm platform for high-throughput preparation and testing. (**c**,**d**) Loading amount distribution with and without GA. (**e**) Mean absolute error of model predictions. (**f**) Machine learning training workflow combining virtual and real data (Reprinted from Ref. [211] under CC BY 4.0; Arrows in each subfigure indicate: (**a**) mapping from composition labels to corresponding property labels; (**b**) sequential workflow from sample preparation to testing).

Machine-learning-assisted materials design should move from concept to practice by integrating high-throughput first-principles calculations with experimental data. A concrete near-term goal is to build a predictive model that maps composition, surface termination, and layer number to nonlinear optical properties such as modulation depth at specific wavelengths (e.g., 3–5 µm). To train such models, researchers can leverage existing open-source materials databases, including the Materials Project (materialsproject.org), NOMAD (nomad-lab.eu), AFLOW (aflowlib.org), and the JARVIS-DFT database (jarvis.nist.gov). For MXene-specific data, and the aNANt MXene database (anant.mrc.iisc.ac.in) provides curated structural and electronic properties. These resources, combined with high-quality experimental nonlinear optical data (e.g., Z-scan results), can be used to train graph neural networks or random forest models to predict performance before synthesis [162,209,210]. Figure 10 illustrates a typical machine-learning-driven workflow combining automated experimentation and active learning [211].

Achieving femtosecond mode-locking in the 3 µm band is a milestone that requires coordinated advances on three fronts. First, on the material side, we recommend systematic exploration of MXenes with smaller bandgaps or stronger mid-IR nonlinearity, such as functionalized Mo_2_Ti_2_C_3_T_x_ or Nb_4_C_3_T_x_, whose modulation depth at 2.8 µm has already reached 40% [53]. Second, device integration should leverage plasmonic enhancement (e.g., Au or Ag nanoparticle decoration) to boost the effective nonlinearity, as demonstrated for V_2_C at 1.55 µm [132]. Third, on the cavity design front, dispersion management using chalcogenide or fluoride fibers with tailored group-velocity dispersion should be implemented. A concrete first target is to demonstrate a mode-locked fiber laser at 2.8 µm with a pulse duration below 500 fs, using a combination of Mo_2_Ti_2_C_3_T_x_ SA and an Er:ZBLAN gain fiber.

On-chip integration should target the development of MXene-based ultrafast pulse sources and modulators on silicon or silicon nitride platforms. A practical pathway is to deposit few-layer MXene films onto the cladding of a silicon nitride waveguide via spin-coating or Langmuir–Blodgett assembly, followed by evanescent field coupling to achieve saturable absorption. The near-term goal should be to demonstrate a mode-locked laser or a pulsed source with a repetition rate > 10 GHz, insertion loss < 2 dB, and modulation depth > 10%, using a hybrid integration approach. Such devices could find immediate applications in on-chip optical interconnects and microwave photonics [209,212].

Multiscale simulation and in situ characterization should be integrated to bridge the gap between atomic-level predictions and device-level performance. On the simulation side, we propose a workflow that couples density functional theory (DFT) calculations of electronic structure with machine-learning potentials to simulate carrier dynamics, followed by solving the nonlinear Schrödinger equation for the mode-locked laser cavity. On the experimental side, operando transient absorption spectroscopy and Raman microscopy under realistic laser pump conditions should be used to monitor the evolution of surface terminations and interlayer spacing during continuous operation. A valuable community effort would be to create a publicly accessible database linking computed nonlinear optical coefficients (e.g., Im[χ^(3)^]) for various MXene compositions to experimentally measured pulse parameters, which could significantly accelerate the design of new SAs [31,193,209].

Industrial-scale fabrication requires a shift from laboratory-scale coating to continuous, high-throughput manufacturing methods. Roll-to-roll printing of MXene-polymer composite films using slot-die or spray coating has already shown promise for large-area production, but the thickness uniformity and defect density must be improved to meet the requirements of photonic devices. A realistic near-term target is to achieve wafer-scale films with thickness variation below 10% and lateral dimensions exceeding 10 cm. Equally important is the development of robust encapsulation schemes that protect MXene SAs from ambient oxidation without compromising optical performance. Multilayer barriers combining polymers (e.g., Parylene or SU-8) with thin inorganic layers such as Al_2_O_3_ deposited by atomic layer deposition have been tested in research settings; the next step is to validate their long-term stability under real laser operating conditions, aiming for a lifetime of several thousand hours. Finally, close collaboration with laser manufacturers is essential to integrate these SAs into commercial systems and to conduct accelerated aging tests that reflect actual field use. Such partnerships would help identify failure modes and optimize the fabrication process for cost-effective, high-yield production [161,212]. The final section summarizes the key conclusions of this review.

## 7. Conclusions

Metallic MXenes, as a new class of two-dimensional SA materials, have demonstrated enormous application potential in ultrafast mode-locked and Q-switched lasers owing to their metallic conductivity, ultrafast carrier relaxation, broadband response, and high damage threshold. From the interdisciplinary perspective of materials engineering and photonic applications, this review has systematically summarized the research progress of MXene-based SAs and draws the following main conclusions.

The structure–property relationship of MXene materials is central to their performance optimization. Their nonlinear optical properties, such as modulation depth, relaxation time, and damage threshold, are intimately correlated with their electronic structure, surface terminations, dimensionality, and interlayer coupling modes. Among these factors, termination engineering and dimensionality control represent the most direct and effective means for tailoring performance, while the new insight concerning interlayer coupling in stacked structures opens up the possibility of simplifying fabrication.

The performance advantages of MXene materials are fully manifested in key metrics. Through cross-comparative analysis, it is shown that MXenes exhibit unique advantages that surpass or rival those of conventional materials in the following aspects: generation of sub-200 fs ultrashort pulses enabled by their ultrafast relaxation characteristics; watt-level high-power and tens-of-microjoule high-energy output owing to their high damage threshold; ultrabroadband response covering the near-infrared to mid-infrared (~3 μm) region; and exploration of novel pulse states such as high-repetition-rate harmonic mode-locking.

Regarding the performance optimization strategies of MXene materials, synergistic optimization and plasmonic enhancement currently represent the research frontiers, as a single strategy can hardly meet all application requirements. A multi-dimensional synergistic strategy that combines termination engineering, heterostructure construction—particularly compositing with metal nanoparticles to exploit plasmon resonance—and composite matrix optimization is the key to comprehensively enhancing the overall performance of MXene SAs and advancing them toward practical applications.

Currently, MXene SAs have already achieved high-performance Q-switched operation in the 3 μm band, but realizing femtosecond mode-locking is the next core objective that urgently needs to be broken through in this field, and mid-infrared mode-locking should be the next milestone breakthrough. This calls for synergistic innovation across materials, devices, and laser physics.

Looking ahead, by addressing the core challenges of standardized preparation, environmental stability, mid-infrared mode-locking, and precise control of carrier dynamics, and by integrating cutting-edge technologies such as machine learning, on-chip integration, and multiscale simulation, MXene-based SAs are expected to play a core role in the next generation of high-performance, integrated, and multi-band ultrafast photonic devices, injecting new vitality into technological innovation in fields such as advanced manufacturing, optical communications, healthcare, and spectroscopy.

## Figures and Tables

**Table 1:** Fundamental physical properties and nonlinear optical parameters of representative metallic MXenes for ultrafast photonic applications.

MXene System	Precursor MAX Phase	Typical Electrical Conductivity (S/cm)	Work Function (eV)	Dominant Surface Termination Tendency	Modulation Depth	Notable Characteristics	References
Ti_3_C_2_T_x_	Ti_3_AlC_2_	1500–24,100	~4.5	mixture of –O, –OH, –F	11.3–33.2% (thickness-dependent, up to ~50% transmittance increase)	most extensively studied, performance benchmark	Ref. [13]: 24,100 S/cm (vacuum-filtered film, four-probe); Ref. [16]: 4600 S/cm (single-flake); Ref. [47]: 11.3–33.2% modulation depth at 1.55 µm, thickness ~50 nm, two-arm detection
Ti_3_CNT_x_	Ti_3_AlCN	5000–10,000	~4.7	predominantly –O, –F	thickness-dependent (quantitative data lacking)	N doping enhances electrical conductivity and stability	Ref. [48]: 5000–10,000 S/cm; modulation depth not systematically quantified
V_2_CT_x_	V_2_AlC	1000–3250	~5.0	predominantly –O, –OH	1.6–50% (wavelength- and structure-dependent)	ultrahigh damage threshold (~70 mJ/cm^2^)	Ref. [43]: 3250 ± 100 S/cm after annealing; Ref. [49]: 1000–3000 S/cm. Modulation depth: 23.7% at 1 µm (Ref. [26]), 1.6% (fiber-integrated) and ~50% (thin-film Z-scan) at 1.55 µm
Nb_2_CT_x_	Nb_2_AlC	500–2000	~4.9	predominantly –O, –OH	~13% (uncoated, at 1.55 μm); up to 88% for composite films	ultrafast relaxation time (~37 fs)	Ref. [45]: 500–800 S/cm; Ref. [50]: up to 2000 S/cm (optimized synthesis). Modulation depth: 13% from Ref. [45]; 88% for Nb_2_C/TiO_2_/PVA composite (Ref. [51])
Ti_2_CT_x_	Ti_2_AlC	1000–5000	~4.3	mixture of –O, –F	4.5% (at 1.06 μm)	zero bandgap, broadband capability 800 nm–2.8 µm	Ref. [52]: 3700 S/cm (multilayer); Ref. [25]: 1000–5000 S/cm. Modulation depth 4.5% from Ref. [25] (1.06 µm, Z-scan)
Mo_2_TiC_2_T_x_	Mo_2_TiAlC_2_	100–614	~5.2	predominantly –O	5.67% (at 1.55 μm)/40% (at 2.8 μm)	high modulation depth (~40% at 2.8 μm)	Ref. [53]: 614 S/cm (vacuum-filtered film). Modulation depth: 5.67% at 1.55 µm (Ref. [54]), 40% at 2.8 µm (Ref. [53])
Ta_4_C_3_T_x_	Ta_4_AlC_3_	~500	~5.5	mixture of –O, –F	—	extremely high third-order polarizability (on the order of 10^−13^ esu)	Ref. [54]: ~500 S/cm. No quantitative modulation depth data publicly available
V_4_C_3_T_x_	V_4_AlC_3_	~800	~5.1	predominantly –O, –OH	—	used for Q-switching in 2.7 μm ZBLAN fiber lasers	Ref. [55]: ~800 S/cm. Modulation depth not reported
Nb_4_C_3_T_x_	Nb_4_AlC_3_	~600	~5.0	predominantly –O, –OH	—	achieved dual-wavelength mode-locking at 1.56/1.93 μm	Ref. [56]: ~600 S/cm. Modulation depth not quantitatively reported

General notes: Electrical conductivity values vary significantly with synthesis method, film thickness, and measurement conditions (four-probe, two-probe, van der Pauw, etc.). The ranges given represent typical literature values. Modulation depth is highly dependent on measurement wavelength, film thickness, integration architecture, and the fitting model. Values from different studies should be compared with caution. “—” indicates that no quantitative modulation depth data are publicly available. For detailed experimental conditions, readers are referred to the original references.

**Table 2:** Comparison of representative properties of MXene versus conventional saturable absorber materials.

Property/Material	MXene(Ti_3_C_2_T_x_) [17,47]	SESAM [3,4]	Graphene [7,8]	Carbon Nanotubes (CNTs) [5,6]	Black Phosphorus (BP) [72]	TMDs (e.g., MoS_2_, WS_2_) [73]
Spectral range	Broadband (visible to mid-IR, ~0.8–3 µm)	Narrow (bandgap-dependent)	Ultrabroad (visible to THz)	Broad (chirality-dependent)	Mid-IR (0.5–4 µm, layer-dependent)	Visible to near-IR (typically <2 µm)
Modulation depth	Moderate to high (11–40%, up to 88% in composites)	Low to moderate (0.5–20%)	Low (<2%)	Low to moderate (1–20%)	Moderate (5–30%, thickness-dependent)	Low to moderate (1–15%)
Recovery time	Ultrafast (sub-ps to few ps, e.g., ~37 fs for Nb_2_C)	Sub-ps to tens of ps	Sub-ps	Sub-ps to tens of ps	Sub-ps to few ps	Sub-ps to tens of ps
Damage threshold	High (e.g., V_2_C ~ 70 mJ/cm^2^)	Moderate	Low	Moderate	Moderate to high (with encapsulation)	Low to moderate
Environmental stability	Moderate (oxidizes in humid air; requires encapsulation)	High	High	High	Poor (rapid degradation in air)	Moderate (some oxidation)
Fabrication complexity	Moderate (etching, exfoliation, scalable)	High (MBE, complex epitaxy)	Moderate (CVD, exfoliation)	Moderate (deposition, alignment)	Moderate (exfoliation, air-sensitive handling)	Moderate (CVD, exfoliation)
Cost	Low to moderate	High	Low	Low	Moderate	Low to moderate
Integration flexibility	High (solution-processable, fiber-compatible)	Low	High	High	Low to moderate (air-sensitivity restricts handling)	High

Table note: Data sources: MXene [17,47] (see also Table 1 and Section 2.2); SESAM [3,4]; graphene [7,8]; CNTs [5,6]; BP [72]; TMDs [73]. All values are representative; exact performance depends on measurement conditions (wavelength, thickness, integration method, etc.).

**Table 3:** Comparative analysis of characteristics and application scenarios of different fiber integration structures.

Integration Configuration	Interaction Strength	Insertion Loss	Mechanical Stability	Thermal Management Capacity	Industrialization Potential	Applicable Scenarios	References
Tapered/micro-nano fiber	High	Low	Moderate	Good	Moderate	Laboratory research, ultrashort pulse generation	[114,115,116,117]
D-shaped fiber	Moderate	Moderate	Excellent	Good	High	High-power and high-stability industrial lasers	[118,119]
Hollow-core fiber	High	Low	Good	Good	Medium to high	Novel structure exploration, applications requiring large modulation depth	[120]
Etched fiber	Moderately high	Low	Good	Fair	Extremely high	Large-scale production, low-cost optical devices	[121,122,123,124]
Fiber ferrule embedded film	Low to moderate	Moderately high	Fair	Fair	Moderate	Rapid performance testing, teaching demonstration	[125]

**Table 4:** Comparison of sub-picosecond/femtosecond pulse performance achieved with MXene-based SAs.

MXene System	Key Features	Central Wavelength (μm)	Pulse Duration (fs)	Core Mechanism	References
Ti_3_C_2_T_x_	Integrated with micro-nano fiber, strong nonlinear absorption	1.55	104	Evanescent field enhanced light-matter interaction	[129,131]
Ti_3_C_2_T_x_	Few-layer structure via liquid-phase exfoliation, abundant –OH terminal groups	1.55	159	Pioneering broadband nonlinear characterization with large modulation depth	[17]
Ti_3_C_2_T_x_	Few-layer applied in all-solid-state lasers	1.05	316	Compatible with high-power solid-state laser platforms	[128]
Ti_3_CNT_x_	Metallic property with nitrogen doping	1.55	660	Feasibility verification of MXenes as SAs	[129]
V_2_C	Localized surface plasmon resonance enhancement	1.55	486	Achieved high-quality mode-locking with a signal-to-noise ratio of up to 92 dB	[132]
Ti_3_C_2_T_x_ (stacked)	Hundreds of stacked layers with a unique interlayer coupling effect	1.91	897	Broke the conventional optimization strategy of few-layer materials and simplified the preparation process	[20,131]
Nb_4_C_3_	Ultra-broadband mode-locking device	1.56/1.93	~1.66 ps/1.62 ps	Dual-band mode-locking realized by a single component	[130]

Note: The 486 fs data for V_2_C are sourced from Reference [132] (a 2025 arXiv preprint), and the conclusions await further confirmation upon formal publication in a peer-reviewed journal.

**Table 5:** Comprehensive comparison of different performance optimization strategies for MXenes.

Optimization Strategy	Regulated Objects	Key Methods	Improvements in SA Performance	Quantitative Standard	Typical Cases	Limitations	References
Terminal Engineering	Density of electronic states, electrical conductivity	Annealing, plasma treatment	Modulation depth ↑, nonlinear coefficient ↑, non-saturable loss ↓, stability ↑	–OH-terminated MXene exhibits stronger nonlinear absorption	–OH-terminated achieves pulses shorter than 200 fs	Difficulty in precise quantitative regulation and poor uniformity in large-area preparation	[40,95,128,129]
Dimensionality Control	Band structure, relaxation time	Ultrasonic fragmentation, gradient centrifugation	Nonlinear coefficient ↑ (QDs), relaxation time ↓, pulse width ↓	0D quantum dots: maximum TA signal of 1.28 ps, pulse duration of 170 fs	Quantum dots generate 150 fs pulses; stacked deliver pulses around 900 fs	Challenges in mass production of quantum dots; underlying stacking mechanisms remain insufficiently explored	[10,129,179]
Heterogeneous and Composite Structure	Interfacial charge transfer, localized field	Van der Waals stacking, chemical synthesis	Broadens response wavelength range, synergistically enhances nonlinearity, reduces saturation intensity	Remarkably promoted dual-band modulation depth and decreased saturation intensity of MXene/Au hybrids at 1.03 μm and 1.55 μm; achieves a modulation depth of 40% at 2.8 μm	Dual-band mode-locking realized by MXene/Au composites; 40% modulation depth obtained based on	Weak interfacial adhesion and complicated fabrication procedures for large-scale production	[46,53,159]
Composite Matrix	Film-forming ability, mechanical stability	Solution blending, spin coating, printing	Scattering loss ↓, mechanical flexibility ↑, environmental stability ↑, processability ↑	MXene/PVA: nonlinear absorption coefficient at 532 nm; MXene/CNC possesses superior dispersion stability	-NaCMC enables high-energy laser output	Restricted thermal conductivity and deteriorated dispersion at high doping concentrations	[162,180,181,182]

**Note:** The arrows (↑/↓) indicate the relative performance level of each integration configuration for a given metric. ↑ denotes better performance (e.g., higher interaction strength, lower insertion loss, better mechanical stability, etc.), while ↓ denotes poorer performance. For metrics without arrows, the performance is moderate or shows no significant difference among the configurations.

## Data Availability

No new data were created or analyzed in this study. Data sharing is not applicable to this article.
